# A mathematical model to estimate the incidence of child wasting in Yemen

**DOI:** 10.1186/s13031-021-00400-6

**Published:** 2021-08-14

**Authors:** Rana A. Hussein, Mark P. Suprenant, Najwa Al-Dheeb, Saul Guerrero, Eleanor Rogers, Fouzia Shafique, Meredith Dyson, Muhammad H. Zaman

**Affiliations:** 1 Departments of Mathematics and Computer Science, Boston University, Boston, MA USA; 2 Department of Biomedical Engineering, Boston University, Boston, MA USA; 3 Howard Hughes Medical Institute, Boston University, Boston, MA USA; 4 UNICEF, Yemen Country Office, Sanaa, Yemen; 5 UNICEF, NYHQ, New York, USA; 6 Independent Consultant, London, UK; 7 UNICEF Yemen Country Office, Amman, Jordan

**Keywords:** Yemen, Acute malnutrition, Wasting, Mathematical modeling, Incidence correction factor, Child health

## Abstract

**Introduction:**

The ongoing civil war in Yemen has severely restricted imports of food and fuel, disrupted livelihoods and displaced millions, worsening already high pre-war levels of food insecurity. Paired with frequent outbreaks of disease and a collapsed health system, this has brought rates of wasting in children under five to the country’s highest recorded levels, which continue to increase as the crisis worsens and aid becomes increasingly limited. In their planning of services to treat and prevent wasting in children, humanitarian agencies rely on a standard calculation to estimate the expected number of cases for the coming year, where incidence is estimated from prevalence and the average duration of an episode of wasting. The average duration of an episode of moderate and severe wasting is currently estimated at 7.5 months—a globally-used value derived from historical cohort studies. Given that incidence varies considerably by context—where food production and availability, treatment coverage and disease rates all vary—a single estimate cannot be applied to all contexts, and especially not a highly unstable crisis setting such as Yemen. While recent studies have aimed to derive context-specific incidence estimates in several countries, little has been done to estimate the incidence of both moderate and severe wasting in Yemen.

**Methods:**

In order to provide context-specific estimates of the average duration of an episode, and resultingly, incidence correction factors for moderate and severe wasting, we have developed a Markov model. Model inputs were estimated using a combination of treatment admission and outcome records compiled by the Yemen Nutrition Cluster, 2018 and 2019 SMART surveys, and other estimates from the literature. The model derived estimates for the governorate of Lahj, Yemen; it was initialized using August 2018 SMART survey prevalence data and run until October 2019—the date of the subsequent SMART survey. Using a process of repeated model calibration, the incidence correction factors for severe wasting and moderate wasting were found, validating the resulting prevalence against the recorded value from the 2019 SMART survey.

**Results:**

The average durations of an episode of moderate and severe wasting were estimated at 4.86 months, for an incidence correction factor *k* of 2.59, and 3.86 months, for an incidence correction factor *k* of 3.11, respectively. It was found that the annual caseload of moderate wasting was 36% higher and the annual caseload of severe wasting 58% higher than the originally-assumed values, estimated with *k* = 1.6.

**Conclusion:**

The model-derived incidence rates, consistent with findings from other contexts that a global incidence correction factor cannot be sufficient, allow for improved, context-specific estimates of the burden of wasting in Yemen. In crisis settings such as Yemen where funding and resources are extremely limited, the model’s outputs holistically capture the burden of wasting in a way that may guide effective decision-making and may help ensure that limited resources are allocated most effectively.

**Supplementary Information:**

The online version contains supplementary material available at 10.1186/s13031-021-00400-6.

## Introduction

Food security crises are closely linked to armed conflict [1]. As a result of both the widespread destruction and devastation that violent conflict brings as well as many of the indirect effects of war which disrupt the daily lives of civilians, conflict creates many of the conditions that drive hunger. War frequently disrupts the food supply—with starvation used as a tactic of war and warring parties deliberately restricting the distribution of food and critical supplies or through the destruction of farms and livestock. In contexts where health services are also extremely limited and much of the health infrastructure destroyed, conflict-affected areas often see high rates of untreated disease, which worsens the risk of undernutrition. Young children are particularly vulnerable to wasting, a rapid deterioration in nutritional status over a short period of time, and often suffer severe and irreversible consequences as a result [2].

In Yemen, where the country’s current hunger crisis is largely the result of warring parties’ deliberate efforts to restrict food, and the war has crippled the economy and disrupted livelihoods, the role of conflict in creating the conditions that drive wasting among children is strikingly clear. The ongoing civil war in Yemen has resulted in what has been called the worst humanitarian crisis in recent history. The latest IPC analysis for Yemen revealed that 13.5 million people (45% of the analyzed population) were facing high levels of acute food insecurity—in IPC Phase 3 or above [3]. Child wasting rates in Yemen are among the highest in the world and continue to increase as the crisis worsens and aid becomes increasingly limited [4]. In some areas of Yemen, it is estimated that more than one in four children suffer from wasting [5]. Untreated wasting can permanently impair a child’s cognitive and physical development and places them at an increased risk of morbidity and mortality; a wasted child is highly vulnerable to severe and recurrent infections [2]. Nutritional interventions to address child wasting are implemented according to the Community-Based Management of Acute Malnutrition (CMAM) Model, the globally endorsed standard for management of acute malnutrition, also known as wasting. The CMAM model aims to reduce mortality and morbidity from wasting by providing early case-finding and effective treatment and by strengthening the local community’s capacity to prevent, identify and manage wasting.

Since the conflict in Yemen began in 2015, humanitarian agencies have scaled up efforts to both prevent, identify and treat cases of Global Acute Malnutrition—Moderate Acute Malnutrition (MAM), also known as moderate wasting, and Severe Acute Malnutrition (SAM), also known as severe wasting– in children under five. (Though the terms wasting and acute malnutrition are often used interchangeably, acute malnutrition is the umbrella term under which wasting falls. Acute malnutrition is defined by the presence of wasting and/or bilateral pitting nutritional oedema [6]. However, the terms moderate and severe wasting will be used throughout this paper to refer to the broader category of acute malnutrition. This is in accordance with the recent shift within the public health and nutrition community towards a generalized use of the term wasting to refer to acute malnutrition as defined by Weight-for-height Z-Score (WHZ), mid-upper arm circumference (MUAC) and/or oedema.)

For planning purposes, humanitarian agencies use prevalence estimates and historical program data to estimate the expected number of cases of moderate and severe wasting as well as expected treatment coverage for the coming year. Knowing the rate of incidence, defined as the number of new cases of wasting which develop over a specified period of time, is critical in anticipating the needs of a program and effectively planning treatment services and resource allocation. However, given that it is difficult to directly observe and measure rates of incidence, estimates of annual wasting caseloads are found using a standard relationship between incidence, prevalence and the average duration of an episode of wasting as described by Eq. 1.

1 $$Incidence =prevalence \times \frac{12}{duration\ of\ episode}$$

Or, expressed in terms of the incidence correction factor *k*:

$$Incidence =prevalence \times k$$

Estimates of the prevalence of wasting are available through cross-sectional SMART surveys conducted annually in Yemen, and the average duration of an untreated episode of moderate and severe wasting has been estimated at 7.5 months (for *k* = 1.6). This approach for incidence estimation has been proposed for use in the CMAM model to estimate under-five wasting caseloads across all contexts [7].

The 7.5-month value was found from two cohort studies conducted in the Democratic Republic of Congo and Senegal in the 1980s and, in the absence of other estimates, is currently used globally [8]. It is difficult to directly observe and estimate the duration of an episode of wasting and so revised, context-specific estimates of the duration of an episode remain limited. (Ethical constraints prevent the possibility of directly measuring the duration of an untreated episode given that this would require following a cohort of wasted children while denying them treatment.) The assertion that a single, standard estimate of the average duration of an untreated episode of wasting is insufficient is supported by both theoretical and quantitative evidence. Intuitively, it would be expected that the average duration of an episode of wasting—a value directly affected by contextual factors such as food availability, disease rates, and treatment accessibility—varies considerably by context. In addition, values derived from the 1980s likely cannot even be applied to the regions from which they were derived today. A number of recent studies have confirmed that this value does in fact vary by context. An analysis of cohort and survey data from three West African countries (Mali, Niger and Burkina Faso) between 2009 and 2012 showed that the incidence correction factor for severe wasting varies widely by country [9]. More recent studies conducted in Mali, Burkina Faso, Niger and Nigeria have reached similar findings [10–12]. In each of these contexts, the 7.5-month value was found to considerably underestimate incidence.

Reliable estimates of caseload are needed for effective service planning and are critical in a context where funding and resources are extremely limited; however, the existing, conventional approach to estimate incidence presents several major limitations. While it is clear that a single k-value cannot be applied to all contexts, little has been done to explore the incidence of wasting in the context of Yemen. Without a context-specific incidence correction factor for Yemen, when Eq. 1 is used to estimate incidence, the only source of variance in this calculation from year to year is prevalence, as measured by the SMART survey. Basing all planning decisions primarily on annual estimates of prevalence—which only captures a snapshot at a single point in time and is a function of not only incidence but also recovery, treatment and fatality rates – provides limited insight that can be used to guide policy decisions. In addition to the fact that wasting incidence has been largely unexplored in the context of Yemen, beyond Yemen, past work which has explored context-specific incidence correction factors has focused primarily on severe wasting; little has been done to explore the incidence of moderate wasting or the relationship between moderate and severe wasting in studies of incidence. With severe wasting arising as existing cases of moderate wasting worsen in severity, an understanding of the interplay between them is needed to holistically assess the burden of wasting. Attempting to measure the incidence of moderate and severe wasting while assessing each one independently of the other neglects the known paths between them and ways in which they inherently interact to form a connected system whereby cases of moderate wasting may progress to severe wasting and cases of severe wasting may improve to moderate wasting.

To address these limitations, we aimed to derive context-specific incidence correction factors for moderate and severe wasting for the governorate of Lahj, Yemen. In doing so, we aimed to model the complete system of interactions which determines the burden of moderate and severe wasting by capturing the bidirectional paths between them. We developed a Markov model to represent this system—a model commonly used in epidemiology to model the progression of disease—and derive estimates of the average duration of an episode (and corresponding incidence correction factors) of moderate and severe wasting. Our model examines the governorate of Lahj, Yemen. Lahj was selected in accordance with guidance from UNICEF team members given the severity of the situation and the relative strength of data reporting from the region given that the area is not one of active conflict.

The model-derived incidence correction factors provide adjusted, context-specific estimates of incidence which may allow for more effective service planning and policy decisions by providing more accurate estimates of the number of new cases expected to develop. Additionally, the model framework provides a tool for burden estimation and planning which simulates ground realities using routinely-collected data and therefore does not require direct observation, as has been the case for several similar studies in other regions which estimated the average duration of an episode through longitudinal cohort studies. The model also seeks to capture the complete system of interactions between rates of incidence, the various stages in the progression of wasting, as well as rates of treatment and their respective outcomes, which collectively determine the burden of wasting. Because the model captures each of these paths, decision-makers can modify the model’s parameters to holistically simulate the long- and short-term consequences of a potential decision, such as scaling up a given intervention, and use the model’s outputs to guide decisions about future interventions.

## Methods

The burden of wasting is determined by a set of paths between moderate wasting, severe wasting and severe wasting with complications as well as treatment admissions and outcomes, creating a network of states for children to move between. This system easily maps onto the general framework of a Markov model, which was used to represent this system. These models are commonly used for probabilistic modeling, especially in epidemiological studies where there are a defined number of outcomes or health states being modeled [13]. To define a Markov model, the following elements are needed: a set of mutually exclusive and exhaustive possible states, the probabilities of initially residing in each of these states, called the initial state distribution, and the probability of transitioning from any one state to every other state, called transition probabilities. Transitions within our model were defined at monthly time intervals. Given the initial state distribution and the set of transition probabilities, the model provides a breakdown of the number of children residing in each state each month.

**Fig. 1:**
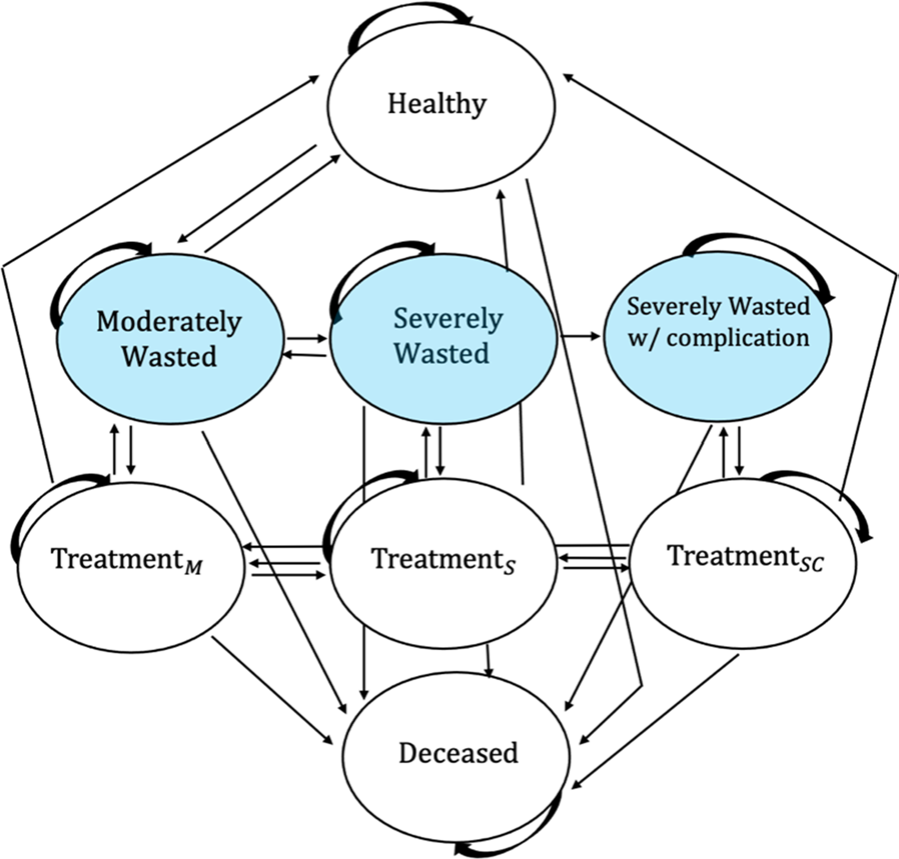
Model State-Transition Diagram. Arrows depict all possible transitions between nutritional states. Blue states indicate a state where a child is wasted but not currently in treatment. ${{\text{Treatment}}_{M, }{\text{Treatment}}}_{S}$ and ${\text{Treatment}}_{SC}$ refer to treatment for moderate wasting, severe wasting, and severe wasting with complications, respectively

A child can be classified as being either non-wasted, wasted but not in treatment, in-treatment for wasting or deceased. The Markov model presented distinguishes between each level of wasting (moderate wasting, severe wasting and severe wasting with complications) and its respective treatment program. It is composed of eight nutritional states: *Healthy, Moderately Wasted, Severely Wasted*, *Severely Wasted with Complications*, *Treatment*_*M*_*, Treatment*_*S*_*, Treatment*_*SC*_ and *Deceased*. If a child resides in either the *Moderately Wasted, Severely Wasted, or Severely Wasted with complications* state, they are wasted but not currently in treatment. Because the model accounts for rates of defaulters (children who stop attending treatment appointments before reaching discharge criteria) and non-responders (children for whom treatment fails), being wasted but not currently in treatment does not imply that the child has never been admitted to treatment; the term *untreated* is therefore not used in the model. During the model’s simulation, children can move between states at a set of monthly transition rates, representing the probability that a child in one state will move to another (or remain in the same one) from one month to the next. All possible transitions are depicted by the arrows in Fig. 1. Given the initial state distribution and the set of transition probabilities, the model provides a breakdown of the number of children residing in each nutritional state each month.

The model is based on the following assumptions:

1. A child must reside in a wasting state (*Moderately Wasted, Severely Wasted or Severely Wasted with complications*) before entering a treatment state.
2. A child cannot transition from the *Health*y state directly to the *Severely Wasted* state; a child residing in the *Severely Wasted* state must have previously resided in the *Moderately Wasted* state.
3. Spontaneous recovery can occur among cases of moderate wasting.
4. A severely wasted child will not return to the *Healthy* state without first residing in either the ${\text{Treatment}}_{S}$ state or the *Moderately Wasted* state. A child cannot transition directly from the *Severely Wasted* state to the *Healthy* state; however, they may transition to *Moderately Wasted* and from *Moderately Wasted*, return to the *Healthy* state.
5. A child in treatment for any form of wasting may default from or fail to respond to treatment, in which case they would transition from the treatment state back to the wasting state (*Moderately Wasted, Severely Wasted, or Severely Wasted with complications*) in which they previously resided.
6. The *Deceased* state is an absorbing state, meaning that once a child enters this state, they cannot transition out of it.

### Determining the initial state distribution

The model’s initial state distribution was informed by 2018 cross-sectional SMART survey data available at the Nutrition Cluster level, reporting the prevalence of moderate and severe wasting among under-five children in Lahj using Weight-For-Height Z-Score as the screening metric [14]. Two separate surveys were conducted in Lahj in August of 2018: one in the highlands region and another in the lowlands region. The two datasets were combined to find the prevalence within the full governorate, using SMART estimates of the under-five population of each region of Lahj and SMART estimates of the prevalence of moderate and severe wasting for each region. Data from the Yemen Nutrition Cluster were used to estimate the average number of children enrolled in treatment for moderate wasting treatment (Targeted Supplementary Feeding Programs, TSFP), treatment for severe wasting (Outpatient Therapeutic Feeding Programs, OTP) and treatment for severe wasting with complications (Therapeutic Feeding Centers, TFC) each month.

While the SMART procedure does not distinguish between wasted children not currently in treatment and those who are enrolled in treatment programs, the model framework did designate two separate states to reflect this distinction, which required that several simplifying assumptions be made. For severe wasting, it was assumed that among the total number of children enrolled in treatment in a given month, only those admitted within the past month would still satisfy the Z-Score used to classify severe wasting cases (Z-Score <  − 3.0). It was therefore assumed that these children, in addition to those who were wasted but not enrolled in treatment, would have been included in the SMART survey’s reported prevalence. Thus, in the model’s initial distribution, children enrolled in treatment for at least one month would be included in the *Treatment*_*s*_ state while those who had been enrolled in treatment for less than one month would be included in the *Severely Wasted* state [6]. The same assumptions were made about cases of moderate wasting (− 3.0 < Z-Score <  − 2.0). In the absence of estimates of the specific prevalence of severe wasting with complications, it was assumed that the ratio of complicated to uncomplicated cases of untreated severe wasting was equivalent to the ratio of uncomplicated treatment admissions to complicated treatment admissions. Because in-patient treatment for complicated severe wasting generally takes less than one month, it was assumed that no children were in treatment for severe wasting with complications to start. The complete initial state distribution is shown in Table 1. Given that the prevalence of wasting varies considerably by season, it was established that the model would begin its simulated year in August—the month during which the 2018 SMART surveys were conducted in Lahj. The model would be run until October 2019, the date of the subsequent SMART survey in 2019, in order to provide a basis for comparison for the resulting prevalence of moderate and severe wasting at the end of its simulation [15].

**Table 1:** Initial state distribution. Number of children in each state and corresponding initial state probability at start of model simulation

Nutritional state	Number of children	Initial state probability
Healthy	129,581	0.7717
Severely Wasted	4199	0.0250
Moderately Wasted	19,224	0.1145
Severely Wasted with Complications	197	0.001174
Treatment_M_	9987	0.05948
Treatment_s_	4719	0.02810
Treatment_SC_	0	0.0
Deceased	0	0.0

### Estimating time-varying probabilities: treatment admission transition probabilities

All transition probabilities representing the probability of a child being admitted to a treatment program (including ${p}_{S{T}_{S}}$, ${p}_{M{T}_{m}}$, and ${p}_{C{T}_{C}}$) were time-varying. Given that monthly treatment admission data was available, and admissions varied considerably from month to month, the respective transition probabilities were recalculated for each month during which the model was run to provide time-varying transition probabilities. Though a deeper analysis of the underlying causes of any significant variations observed in each month’s recorded admissions would allow the model to operate with a predictive capacity, because the model aimed to retrospectively estimate incidence, time-dependent probabilities were directly estimated to reflect these variations without further consideration of their causes.

### Estimating stationary transition probabilities: treatment outcomes, general mortality, spontaneous recovery, untreated case fatality

All other transition probabilities were computed as stationary probabilties, meaning they remain unchanged over time. Treatment outcome probabilties (with outcomes including cure, default, non-response, referral to other program, and in-treatment fatality) were estimated from monthly Yemen nutrition cluster data provided by the UNICEF Yemen country office. This data included complete records of rates of admission and respective outcomes of children enrolled in TSFPs for moderating wasting treatment, OTPs for severe wasting treatment and TFCs for complicated severe wasting treatment for all CMAM interventions implemented across the governorate.

The general mortality rate ${(p}_{HD})$ was estimated using the under-five mortality rate recorded in the Lahj 2019 SMART surveys [15]. Given both the scarcity of data coming from Yemen and the general ethical constraints of monitoring outcomes of untreated wasting, several model parameters were informed by studies from comparable contexts. The model’s untreated moderate and severe wasting case fatality rates (${p}_{MD, } {p}_{SD}, { p}_{CD}$) were derived from hazard ratios estimated by a pooled meta-analysis using data from cohorts across different contexts before the onset of CMAM [16]. Both transition probabilities representing a transition of recovery (${p}_{MH, } {p}_{SM}$) were also derived from studies from other contexts [17, 18]. The rate of spontaneous recovery for moderate wasting was selected from a systematic review by Lelijveld et al. in which they examine a number of studies examining various forms of treatment for moderate wasting [19]. Among those included, the randomized controlled trial from Burkina Faso was selected for use in the model given that it was the only one where the control group was not provided micronutrient treatment (and this transition probability needed to reflect outcomes of untreated wasting), where the setting was not food secure, and where study definitions aligned with current definitions of moderate wasting [18]. The probability of a child improving from severely wasted to moderately wasted was estimated from a single time point follow-up study of severely wasted children in India [17]. All transition probabilities, as well as their conceptual interpretation and respective sources, are summarized in Table 2.

**Table 2:** List of Model’s Transition Probabilities and Respective Conceptual Interpretations and Sources. Each transition probability represents the probability that a child in a given nutritional state moves to another within one month

Nutrition state transition	Interpretation	Transition probability	Source data/notes
*From Healthy state*			
${Healthy\ to\ Healthy\ (p}_{HH})$	Remains healthy	1–${p}_{HM} - 0.000750$	–
${Healthy\ to\ Moderately\ Wasted\ (p}_{HM})$	Incidence of Moderate Wasting	Unknown	To be estimated
${Healthy\ to\ Severely\ Wasted\ (p}_{HS}$)	Develops severe wasting from healthy	0	Model assumption 2
${Healthy\ to\ Severly\ Wasted\ with\ complications\ (p}_{HC})$	Develops severe wasting w/ complication from healthy	0	–
${Healthy\ to\ Deceased\ (p}_{HD})$	General Under-five Mortality Rate	0.000750	2019 Lahj SMART Survey
*FromSeverely Wastedstate*			
${Severely Wasted to Healthy (p}_{SH})$	Severe Wasting Spontaneous Recovery	0	Model assumption 4
${Severely\ Wasted\ to\ Moderately\ Wasted\ (p}_{SM})$	Untreated severe wasting improves to moderate wasting	0.0914	Single time point follow-up of severely wasted children – India (Sachdev et al.) [17]
${Severely\ Wasted\ to\ Severely\ Wasted\ (p}_{SS})$	Severe wasting remains severe wasting	1−${p}_{S{T}_{S}}-0.0724$	–
${Severly\ Wasted\ to\ Severely\ Wasted\ with\ complications\ (p}_{SC})$	Untreated severe wasting develops medical complication	0.01026	Nutrition Cluster OTP/TFC Data
${Severely\ Wasted\ to\ {{\text{Treatm}}{\text{ent}}}_{S}\ (p}_{S{T}_{S}})$	Admitted to OTP	[0.200,0.678]	Nutrition Cluster OTP Data
${Severely\ Wasted\ to\ Deceased\ (p}_{SD})$	Untreated Severe Wasting Case Fatality	0.00872	Hazard ratios from pooled analysis (Olofin et al.) [16]
*FromModerately WastedState*			
${Moderately\ Wasted\ to\ Healthy\ (p}_{MH})$	Moderate Wasting Spontaneous Recovery	0.07814	Randomized controlled trial—Burkina Faso (Nikièma et al.) [18]
${Moderately\ Wasted\ to\ Severely\ Wasted\ (p}_{MS})$	Untreated moderate wasting progresses to severe wasting (severe wasting incidence)	Unknown	To be estimated
${Moderately\ Wasted\ to\ Moderately\ Wasted\ (p}_{MM})$	Moderate wasting remains moderate wasting	1−${p}_{MS}$−${p}_{M{T}_{m}}$−$0.0807$	–
${Moderately\ Wasted\ to\ {\text{Treatment}}_{M}\ (p}_{M{T}_{m}})$	Admitted to TSFP	[0.0529,0.176]	Nutrition Cluster TSFP Data
${Moderately\ Wasted\ to\ Deceased\ (p}_{MD})$	Untreated Moderate Wasting Case Fatality	0.00260	Hazard ratios from pooled analysis (Olofin et al.) [16]
*From Severely Wasted with complications state*			
${Severely\ Wasted\ with\ complications\ to\ Severely\ Wasted\ with\ complications\ (p}_{CC})$	Severely wasted w/ complications remains severely wasted w/ complications	1−${p}_{C{T}_{C}}-$0.00260	–
${Severely\ Wasted\ with\ complications\ to\ {\text{Treatment}}_{SC}\ (p}_{C{T}_{C}})$	Admitted to TFC	[0.0,0.229]	Nutrition Cluster TFC Data
${Severely\ Wasted\ with\ complications\ to\ Deceased\ (p}_{CD})$	Severely Wasted w/ complications Untreated Case Fatality	0.00260	Hazard ratios from pooled analysis (Olofin et al.) [16]
*From*${\text{Treatment}}_{S}$*State*			
${{\text{Treatment}}_{S}\ to\ Healthy\ (p}_{{T}_{S}H})$	$\text{Cured at OTP}$	0.307	Nutrition Cluster OTP Data
${{\text{Treatment}}_{S}\ to\ Severely\ Wasted\ (p}_{{T}_{S}S})$	Defaults from OTP	0.0262	Nutrition Cluster OTP Data
${{\text{Treatment}}_{S}\ to\ {\text{Treatment}}_{M}\ (p}_{{T}_{s}{T}_{m}})$	$\text{Referred from OTP to TSFP}$	0.00503	Nutrition Cluster OTP Data
${{\text{Treatment}}_{S}\ to\ {\text{Treatment}}_{S}\ (p}_{{T}_{s}{T}_{S}})$	Remains in OTP	0.657	Nutrition Cluster OTP Data
${{\text{Treatment}}_{S}\ to\ {\text{Treatment}}_{SC}\ (p}_{{T}_{s}{T}_{C}})$	Referred from OTP to TFC	0.004204	Nutrition Cluster OTP Data
${{\text{Treatment}}_{S}\ to\ Deceased\ (p}_{{T}_{S}D})$	In-treatment (OTP) case fatality	0.000156	Nutrition Cluster OTP Data
*From*${\text{Treatment}}_{M}$*State*			
${{\text{Treatment}}_{M}\ to\ Healthy\ (p}_{{T}_{m}H})$	Cured at TSFP	0.103	Nutrition Cluster TSFP Data
${{\text{Treatment}}_{M}\ to\ Moderately\ Wasted\ (p}_{{T}_{m}M})$	Default from TSFP	0.00442	Nutrition Cluster TSFP Data
${{\text{Treatment}}_{M}\ to\ {\text{Treatment}}_{M}\ (p}_{{T}_{m}{T}_{m}})$	Remains in TSFP	0.892	Nutrition Cluster TSFP Data
${{\text{Treatment}}_{M}\ to\ {\text{Treatment}}_{S}\ (p}_{{T}_{m}{T}_{S}})$	Referred to OTP from TSFP	0.000918	Nutrition Cluster TSFP Data
${{\text{Treatment}}_{M}\ to\ Deceased\ (p}_{{T}_{m}D})$	In-treatment (TSFP) case fatality	0	Nutrition Cluster TSFP Data
*From*${\text{Treatment}}_{SC}$*State*			
${{\text{Treatment}}_{SC}\ to\ Healthy\ (p}_{{T}_{C}H})$	Cured at TFC	0.792	Nutrition Cluster TFC Data
${{\text{Treatment}}_{SC}\ to\ {\text{Treatment}}_{M}\ (p}_{{T}_{C}{T}_{m}})$	Referred from TFC to TSFP	0.0492	Nutrition Cluster TFC Data
${{\text{Treatment}}_{SC}\ to\ {\text{Treatment}}_{S}\ (p}_{{T}_{C}{T}_{S}})$	Referred from TFC to OTP	0.158	Nutrition Cluster TFC Data
${{\text{Treatment}}_{SC}\ to\ {\text{Treatment}}_{SC}\ (p}_{{T}_{C}{T}_{C}})$	Remains in TFC	0	Nutrition Cluster TFC Data
${{\text{Treatment}}_{SC}\ to\ Deceased\ (p}_{{T}_{c}D})$	In-treatment (TFC) case fatality	0	Nutrition Cluster TFC Data
*FromDeceasedstate*			
${Deceased\ to\ Deceased\ (p}_{DD})$	*Deceased* state is absorbing	1	Model assumption 6

### Estimating average duration of an episode of severe wasting

**Fig. 2:**
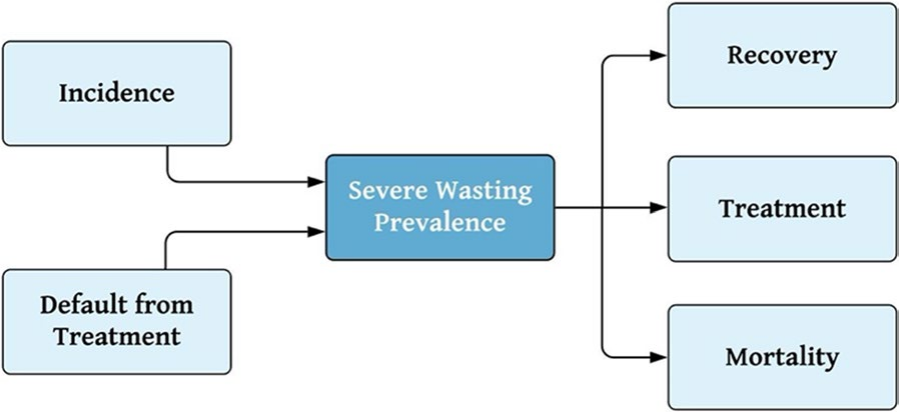
Magnified view of rates determining the prevalence of severe wasting within model framework. In the model framework, the prevalence of severe wasting can be conceptualized as a system of inflows, paths through which children enter the prevalence pool, and outflows, paths through which children leave the prevalence pool. Within this system, the incidence of severe wasting was the single unknown. Repeated model calibration was used to estimate the rate of incidence of severe wasting

With all other transition rates calculated, the incidence of severe and moderate wasting was found using a process of repeated model calibration. As shown in Fig. 2, the prevalence of severe wasting can be understood as a function of several other model rates, including those determining the probability that a child enters the *severely wasted* state—incidence or default from treatment—and those determining that a probability that a child leaves the *severely wasted* state—through recovery, treatment or mortality. Thus, in terms of the model’s states, children can leave the *Severely Wasted* state either by entering the ${\text{Treatment}}_{S}$ state, entering the *Moderately Wasted* state (recovery) or entering the *Deceased* state. Children enter the *Severely Wasted* state when they develop severe wasting from moderate wasting, representing the rate of incidence, or transition from ${\text{Treatment}}_{S}$ back to *Severely Wasted* (default.) Because the prevalence of moderate wasting is directly influenced by the incidence of severe wasting, the incidence of severe wasting needed to first be estimated, before being used to inform the estimation of moderate wasting prevalence.

Given that the incidence of severe wasting was the single unknown in Fig. 2, while each of the other transition probabilities as well as the expected prevalence was known, incidence was back-calculated through model calibration. Using the initial state distribution informed by 2018 data shown in Table 1 as well as the model’s established non-incidence rates, the model was run for 14 simulated months (until October 2019) and calibrated with a range of estimates of severe wasting incidence, in order to find the value which would result in a prevalence that matched the value reported in the 2019 SMART survey at the end of the model’s simulated period [15]. At the end of the simulated period, the number of children in the model’s *Severely Wasted* state would represent the prevalence of untreated severe wasting. To remain consistent with the original assumptions about the combined prevalence of untreated and currently-in-treatment cases represented by the SMART prevalence, the estimated prevalence used for validation against SMART results was calculated as the sum of the number of children in the *Severely Wasted* state at month 14 (untreated case prevalence) and the number of children admitted to treatment within the past month.

### Estimating average duration of an episode of moderate wasting

**Fig. 3:**
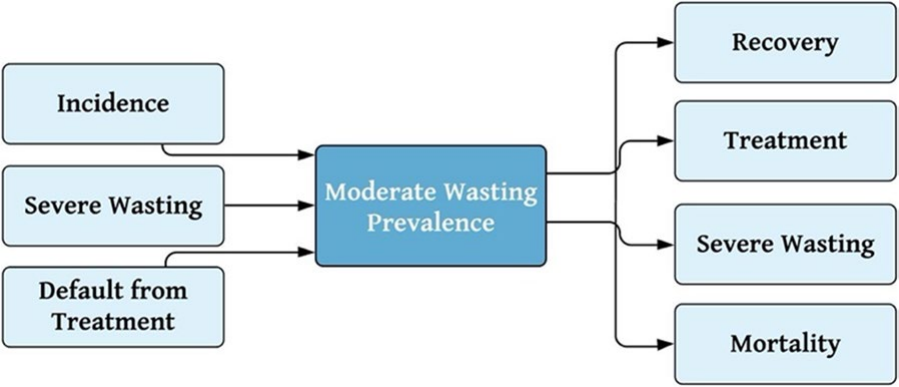
Magnified view of rates determining the prevalence of moderate wasting within model framework. In the model framework, the prevalence of moderate wasting can be conceptualized as a system of inflows, paths through which children enter the prevalence pool, and outflows, paths through which children leave the prevalence pool. Upon estimating the incidence of severe wasting, the incidence of moderate wasting remained the single unknown with this system and was estimated through repeated model calibration

Model calibration was also used to estimate the average duration of a moderate wasting episode. Because the model framework assumes all severe wasting cases develop from existing cases of moderate wasting, the prevalence of moderate wasting is also directly affected by the incidence of severe wasting. The model-derived incidence rate of severe wasting was therefore used as an input in the process of determining the average duration of an episode of moderate wasting. Spontaneous recovery was also assumed to occur among cases of moderate wasting, where a child with moderate wasting but not currently in treatment can return directly to the *Healthy* state. It was also assumed that cases of severe wasting could improve to moderate wasting, contributing an additional path of entry into the *Moderately Wasted* state. Using each of the transition probabilities (either entering or leaving the *Moderately Wasted* state) shown in Fig. 3, the model was calibrated to determine the average duration of an episode of moderate wasting, again using the prevalence recorded in the 2019 SMART survey as the basis of comparison. The same assumptions were made about currently-in-treatment and untreated cases as previously described for severe wasting.

### Sensitivity analysis

In order to quantify uncertainty within the model—either resulting from the general uncertainty within program records or the use of several data sources from contexts outside of Yemen—a one-way, deterministic sensitivity analysis was conducted by putting each of the transition probabilities through a range of possible values while holding all others constant and examining the resulting effect on the estimated number of incident cases [20].This was performed for moderate and severe wasting incidence, respectively. Each transition probability directly affecting the incidence of severe wasting—including that of treatment admissions, spontaneous recovery, case fatality, default from treatment and mortality—was allowed to vary between 50 and 150% of its base value. The same analysis was performed for each transition probability directly affecting the incidence of moderate wasting—including severe wasting incidence, treatment admissions, recovery, default from treatment and case fatality. Upon modulating each parameter through the defined range, the same process of model calibration was performed in order to produce the corresponding incidence rate. This analysis would reveal which of these rates had the greatest impact on the incidence rate derived by the model. All analyses were conducted using Python 3.7 and the NumPy library [21].

## Results

### Average duration of episode and context-specific K-values

Upon repeated model calibration, it was found that the average duration of an episode of severe wasting was 3.86 months, with a corresponding incidence correction factor *k* of 3.11. This *k*-value was then used to estimate the incidence of severe wasting (the *Moderately Wasted* to *Severely Wasted* transition probability), and through subsequent model calibration, the average duration of an episode of moderate wasting was estimated at 4.64 months, for an incidence correction factor *k* of 2.59. A comparison of the original episode durations, incidence correction factors and annual caseloads and the respective context-specific values is presented in Table 3. Caseload was defined as the number of prevalent cases at the start of the year plus the number of incident cases over the course of the year. As expected, both values of the average duration of an episode of wasting were considerably lower than the originally assumed value of 7.5 months, meaning the original approach significantly underestimated caseload. The context-specific annual caseloads for moderate and severe wasting were found to be 36% and 58% higher, respectively, than the originally assumed values.

**Table 3:** Estimated episode durations, incidence corrections factors, and annual caseloads for moderate and severe wasting. Original caseload refers to the value estimated with k = 1.6 and context-specific caseload refers to the value estimated using the model-derived k-value

	Original average duration of episode (months)	Context-specific average duration of episode (months)	Original k-value	Context-specific k-value	Original annual caseload	Context-specific annual caseload
Moderate wasting	7.5	4.64	1.6	2.59	49,981	67,891
Severe wasting	7.5	3.86	1.6	3.11	10,919	17,256

Table 4 presents a comparison of the number of children moderately and severely wasted in October 2019 estimated by the model with the number estimated from the October 2019 SMART survey, which was used for validation. The model-derived incidence rates could not be directly validated given that the model used the available empiric data to estimate these previously unknown values; the model aimed to utilize the available data regarding outcomes that are directly observable in order to provide information about outcomes which are not.

**Table 4:** Validation of resulting number of children moderately and severely wasted in October 2019 using context-specific incidence rates

	SMART 2019	Model output
Number of children moderately wasted	3733	3738.39
Number of children severely wasted	21,358	21,374.55

Upon running the model between the period of August 2018 and October 2019 using the context-specific incidence rates, the resulting number of children who were moderately or severely wasted in October 2019 were validated against the respective values recorded in the October 2019 SMART survey

**Fig. 4:**
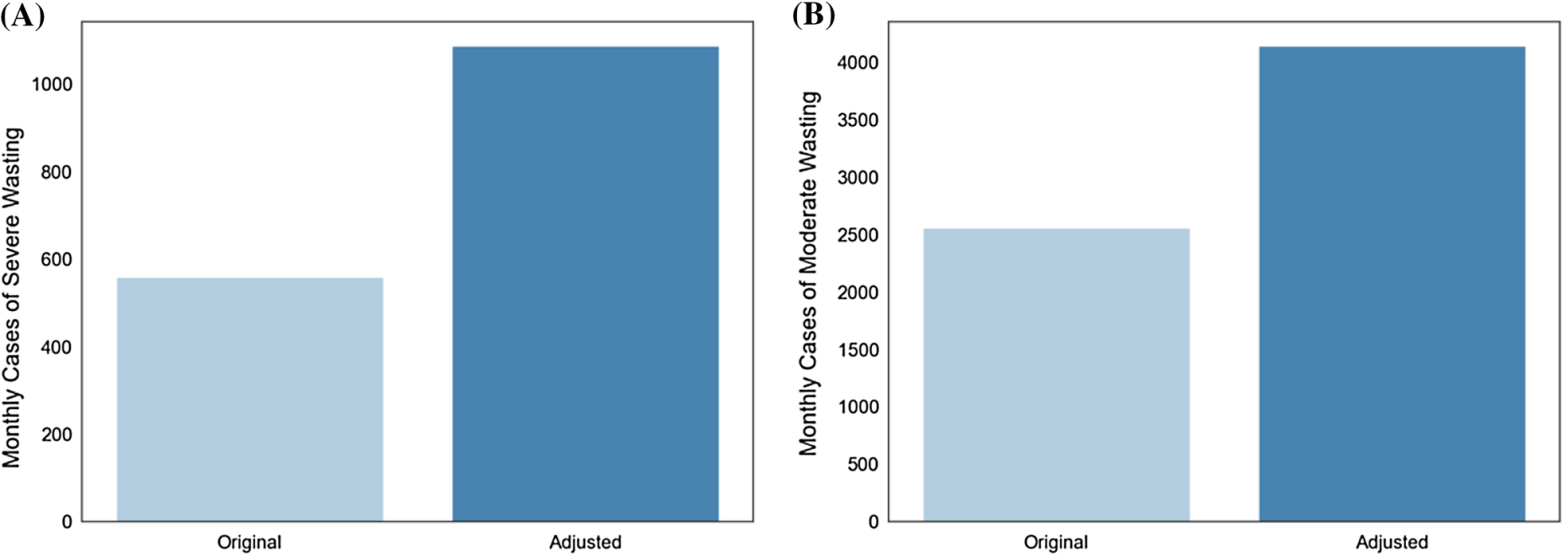
**a** Comparison of estimated monthly number of new cases of severe wasting using original approach (with k = 1.6) and context-specific (model-derived) monthly number of new cases of severe wasting (with k = 3.11). **b** Comparison of estimated monthly number of cases of moderate wasting using original approach (with k = 1.6) and adjusted (model-derived) monthly number of cases of moderate wasting (with k = 2.59)

A comparison of the monthly number of incident cases of moderate and severe wasting is shown in Fig. 4. Knowing the incidence of both moderate and severe wasting—where it was assumed all severe wasting cases developed from existing cases of moderate wasting—the frequency at which cases of moderate wasting progressed to severe wasting could be derived. It was found that approximately 27% of children who were moderately wasted developed severe wasting over the course of the model’s simulation.

### Sensitivity analysis

While the model-derived incidence rates could not be directly validated, the uncertainty within the data used to estimate these values could be quantified by examining the effect of varying model input values (transition probabilities) on model-derived outcome estimates of wasting incidence. The detailed results of these sensitivity analyses are presented in Additional file 1. For both moderate and severe wasting, model-derived estimates of incidence were most sensitive to variation in the transition probabilities for treatment admission and for recovery.

## Discussion

In this manuscript, we provide context-specific estimates of incidence for moderate and severe wasting among under-five children in Lahj, Yemen. We also provide a framework for holistically assessing the burden of wasting which considers the complete system of bidirectional paths which determine the prevalence and incidence of wasting. Accurate estimates of the incidence of wasting are critical for projecting the needs of a program and planning accordingly. Although data from cross-sectional prevalence surveys such as SMART are available, these estimates provide only a snapshot of the situation at a single point in time. By basing assessments on prevalence alone, decision-makers must rely on a metric which varies seasonally and is also dependent on rates of mortality, recovery and treatment coverage. Estimates of monthly incidence, presenting the number of children developing new cases of moderate and severe wasting who will therefore require treatment, provide insights that may be of practical use to decision-makers in their planning.

The model-derived incidence rates align with the consensus within the literature that a single incidence correction factor of 1.6 cannot be sufficient and underestimates caseload, leaving populations of children in need of treatment unaccounted for. Relying on incidence estimates that are not context-specific to guide service planning may lead to potential shortages of program inputs. To the authors’ knowledge, other studies have not explored the incidence of moderate and severe wasting in Yemen, so our results cannot be assessed against other comparable findings. However, qualitative evidence from Yemen further confirms that previous estimates of caseload were considerable underestimates. First, historical program data from Yemen have shown that when caseload is calculated using *k* = 1.6, the number of children admitted to treatment sometimes surpasses the expected monthly caseload, leading to coverage estimates over 100%. Additionally, when the incidence correction factor is assumed to remain unchanged from year to year, the only source of variance in the annual caseload calculation comes from cross-sectional prevalence data. Because of this, estimated caseloads for wasting in Yemen over the last 3 years have remained relatively stable; this does not align with the general instability caused by the conflict or extensive reports showing that the nutrition situation has continued to deteriorate over the past years.

Several studies, using data from cohort studies in Mali, Burkina Faso, Niger and Nigeria, have found context-specific incidence correction factors [10–12]. All found the estimated duration of 7.5 months resulted in underestimates of caseload. An analysis of cohort and survey data from three West African countries (Mali, Niger and Burkina Faso) between 2009 and 2012 showed that the incidence correction factor varies widely by country [9]. In each of these contexts, using a k-value of 1.6 was found to considerably underestimate incidence. As the authors of these works note, these results are not intended to be generalized to other regions where several contextual factors differ greatly. Thus, our results cannot be compared to those found in other contexts. They do, however, confirm the assumption that incidence correction factors vary considerably by context, and a single estimate cannot be appropriate. While it is known that seasonal variations will affect incidence rates of wasting, possible approaches for accounting for seasonal variability have not been extensively explored [22]. Each of the aforementioned studies assumes a constant rate of incidence, though recognizing the limitations of this assumption. Our model’s ability to assess changes in incidence over time is constrained by the frequency at which prevalence data are collected—in this case, once a year. Thus, it can retrospectively provide incidence estimates on an annual basis, which may provide a basis for understanding changes to incidence on a longer scale. If more frequent cross-sectional prevalence data are available in other contexts, the model may be used to provide estimates of incidence over shorter periods of time and thus reflect changes to incidence throughout the year.

While several other studies have aimed to estimate context-specific incidence correction factors in other contexts, the majority have examined severe wasting, and little has been done to explore context-specific incidence rates in Yemen. To the authors’ knowledge, little work has been done to examine the incidence of moderate and severe wasting together, under the same framework. Rather than considering the development of moderate wasting, severe wasting and severe wasting with complications independently of each other, the model framework allows for a consideration of the complete system of interactions that they form. With more accurate estimates of the incidence of severe wasting, the model captures the rate at which moderate wasting is expected to develop into severe wasting. Deriving this information would generally require direct observation of a cohort of moderately wasting children. Additionally, by representing the burden of wasting in a model of this form, the model can not only estimate expected caseload, but also be used as a tool to simulate various scenarios in order to guide planning decisions. For example, by adjusting the rate of treatment for moderate wasting and running the model for several months, decision-makers can understand both the long- and short-term effects of a scale-up of a given treatment program. This would allow them to understand the effects of such a decision on not just the burden of moderate wasting, but also the burden of severe wasting and severe wasting with complications in the long-term. These insights may form the basis of further cost-effective analyses of various programs. Thus, accurate estimates of incidence are not only critical in determining the number of children in need, but also in more holistically assessing the burden of malnutrition.

The model’s outputs may provide a number of insights about program coverage. An alternative, simple approach to adjusting incidence estimates could entail using expected program coverage and treatment admissions to find the total number of treated and untreated cases of wasting, but doing so would assume confidence in current coverage estimates. While coverage may be estimated directly by representative sampling, such estimates remain scarce. Given that a key unknown of the coverage calculation is the number of untreated cases, our model provides a means of estimating this—representing the total number of developing cases which will require treatment—which does not rely on an existing coverage estimate, but which may instead be used to inform more accurate estimates of program coverage.

As is the case with many mathematical models seeking to capture complex processes, our model had several limitations. First, a fundamental property of Markov models is that of “memorylessness”—the assumption that the future states depend only on the current state and not any past states [13]. This assumption limits the model’s ability to capture the way in which a child’s past nutritional status may affect their future outcomes. It is known, for example, that a child who spontaneously recovers from an episode of moderate wasting is at greater risk of developing moderate wasting again, but the model does not account for this. It is possible to introduce a level of consideration of past states while maintaining the Markovian property of memorylessness, by formulating the system as a higher-order Markov model. In this model, rather than expressing the probability of transitioning to a particular state given only the current state, transition probabilities represent the probability of transitioning to a particular state given a particular sequence of past states [23]. This would allow the model to capture the fact that the risk of becoming wasted for a healthy child who had never experienced wasting would differ from that of a child who had previously experienced wasting but later recovered. Our model was not represented this way because quantitative information about these particular outcomes among children in Yemen, such as the probability of becoming wasted again upon recovery, was not available. Given that we did not aim to examine these cases at such an individual level and that rates of default and spontaneous recovery were relatively low, the effects of these limitations on the results were likely minimal. Though the transition probabilities corresponding to treatment admission were time-varying, the assumption that other rates would remain constant presented several limitations. It is likely that mortality rates vary seasonally; however, given the ethical constraints of following a cohort of untreated children, little is known about outcomes of untreated wasting. Existing estimates of untreated case fatality rates for moderate and severe wasting were used for this model which were expressed as a constant rate; given that this data is limited, variations in these rates could not be explored. Among the most uncertain of the model’s transition probabilities are those of spontaneous recovery from moderate and severe wasting. Spontaneous recovery happens unpredictably and is dependent on many contextual factors, with the only empiric data available coming from studies conducted in other contexts and before the introduction of CMAM. Nonetheless, our sensitivity analyses showed that varying these values would by a factor of 50%—150% would never result in an average episode duration as high as 7.5 months. Moreover, treatment admission transition probabilities were shown to have the greatest impact on computed incidence rates, and these were estimated from Yemen-specific data, providing further confidence in the model’s findings.

Several limitations were presented by the limited data available for validation. SMART survey results were a primary source of validation of the model’s results. Because SMART relies on representative sampling in order to estimate prevalence for the entire governorate, a level of uncertainty is expected within its results. The 2018 SMART survey also notes that settlements for internally displaced people (IDPs) were excluded from the sampling frame [14]. It is known that internally displaced people in Yemen face comparatively higher levels of food insecurity and lower levels of access to health and basic services, meaning the survey’s results likely underestimate the prevalence of wasting by excluding these settlements [24]. Additionally, because SMART surveys in Yemen are conducted annually, the absence of intermediate data points meant that monthly prevalence at each iteration of the model could not be validated. Given that conducting widescale cross-sectional prevalence surveys is costly and humanitarian agencies face increasingly limited funding, it is expected that this data will be scarce. Despite this, the model aims to make use of and supplement the available data to offer new insights, improve upon the existing approach for estimating wasting and strengthen understandings of the burden of wasting in Yemen.

Despite its limitations, our model can provide decision-makers with important insights about the expected burden of wasting. Additionally, the model’s ability to holistically capture all determinants of the monthly prevalence of wasting may provide a potential alternative to conducting in-person cross-sectional surveys such as SMART, allowing humanitarian agencies to direct efforts and funds elsewhere. Future work may entail extending the model framework to other conflict-effected settings in order to produce more accurate caseload estimates and consider the expected instability of conflict settings. This may serve to validate the utility and generalizability of the model in other contexts. Future work may also entail building upon the model to explore seasonal changes in the incidence of wasting by considering the direct and indirect drivers of wasting. Incorporating these factors would allow the model to operate with a predictive capacity; by capturing relationships between the incidence of wasting and its underlying causes, the model may anticipate how a change in ground realities may result in a change in the incidence of wasting. The adjusted incidence rates provide a more context-specific improvement from the standard, global estimates, but this approach also assumes the previous year’s caseload can be used to anticipate caseload for the following year. Capturing the determinants of wasting within the model framework would further improve upon this approach by allowing incidence estimates to reflect changes on the ground.

## Conclusion

In this manuscript, we present context-specific estimates for the incidence of moderate and severe wasting in Lahj, Yemen. Accurate estimates of incidence are critical in anticipating program needs and holistically assessing the burden of wasting among children. Confirming the assertion that a single incidence correction factor cannot be sufficient, our results show that previous estimates led to considerable underestimates of caseload and left entire populations of wasted children unaccounted for. In addition to providing improved estimates of caseload, the model may also be used as a decision-making tool, allowing users to modify its parameters to understand the long- and short-term implications of a given interventional decision, which may be used to guide future cost-effectiveness analysis. Additionally, by seeking to estimate the total number of cases of wasting—both treated and untreated—the model provides a basis for providing improved estimates of program coverage. In crisis settings such as Yemen where funding and resources are extremely limited, the model’s outputs may help ensure that limited resources are allocated most effectively and holistically capture the burden of wasting in a way that can facilitate effective decision-making and intervention strategies.

## Supplementary Information

**Additional file 1**. Results of Model Sensitivity Analysis.

## Data Availability

The datasets analyzed during the current study are available in the Nutrition Cluster repository, https://www.humanitarianresponse.info/en/operations/yemen/nutrition

## Abbreviations

CMAM: Community-based Management of Acute Malnutrition
MAM: Moderate acute malnutrition
SAM: Severe acute malnutrition
SMART: Standardized monitoring and assessment of relief and transitions
OTP: Outpatient therapeutic feeding program
TFC: Therapeutic Feeding Center
TSFP: Targeted Supplementary Feeding Program
IPC: Integrated Food Security Phase Classification
