# Supplementary material for: A mathematical model to estimate the incidence of child wasting in Yemen
Source: Confl Health. 2021 Aug 14;15:62. doi: 10.1186/s13031-021-00400-6 (PMC8364017; doi:10.1186/s13031-021-00400-6)
Supplement: Supplementary file 1 — Additional file 1. Results of Model Sensitivity Analysis. [file 13031_2021_400_MOESM1_ESM.docx]

Additional File 1. Supplementary Results.

**Sensitivity Analysis**

While the model-derived incidence rates could not be directly validated, the uncertainty within the data used to estimate these values, could be quantified through sensitivity analysis. The results of the sensitivity analysis for moderate and severe wasting incidence are shown in Figure S1. Longer bars indicate that the corresponding parameter had a greater impact on the resulting incidence, expressed as the monthly number of new cases of moderate and severe wasting. For both moderate and severe wasting, transition probabilities for treatment admissions and spontaneous recovery were those the model was most sensitive to. Bars corresponding to the transition probability for defaulting appear inverted in comparison to the others due to the fact that an increase in defaulting, (and subsequent return to the untreated wasting state) where all other rates and prevalence were held constant, would result in a lower incidence rate. The same is true of the bar corresponding to severe wasting recovery in Figure 5B; an increase in the probability of severe wasting recovery would indicate a higher probability of return back to the *Moderately Wasted* state. The range of values swept for each parameter as well as the resulting k-value is presented in Table S1.

(A)


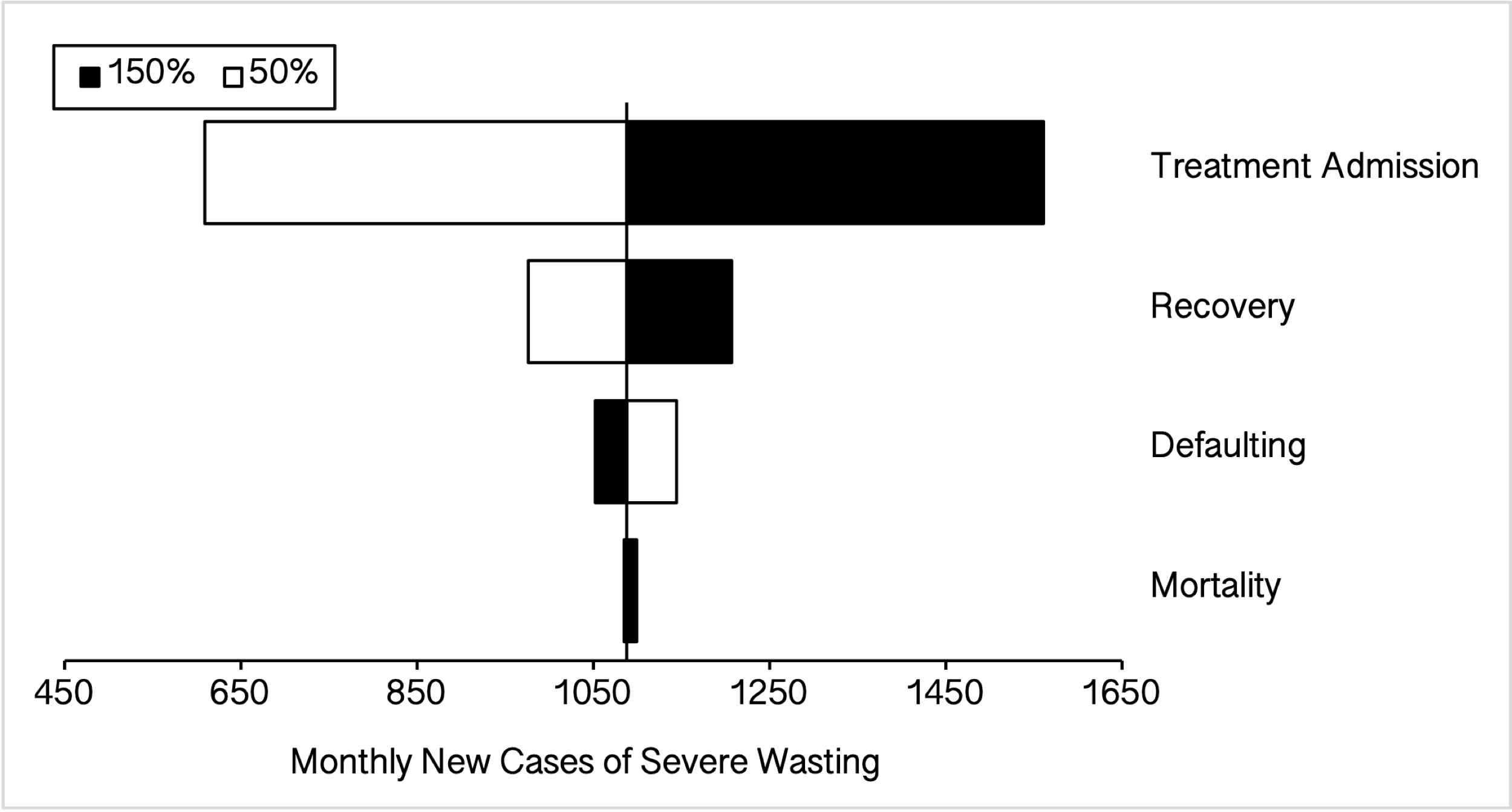


(B)


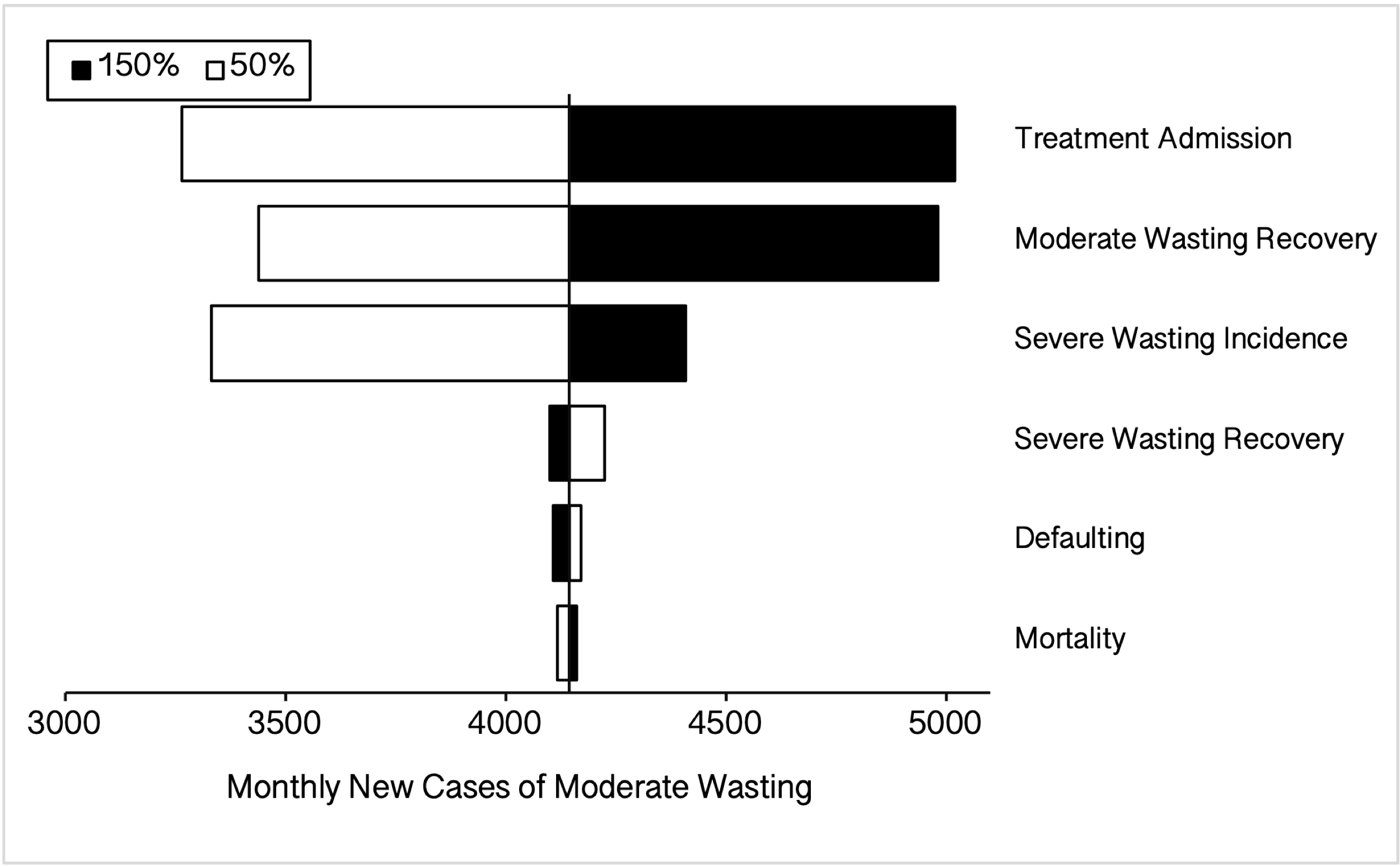


**Figure S1.** Effects of varying the transition probabilities used in the model on the estimated monthly incident cases of (A) severe wasting and (B) moderate wasting.

(A)

|  | **Range Swept** | **Resulting Average Duration (months)** |
| --- | --- | --- |
| **Treatment Admission** | Time-varying | 2.69 – 6.89 |
| **Recovery** | 0.0457 – 0.137 | 3.48 – 4.30 |
| **Defaulting** | 0.0131 – 0.0393 | 3.67 – 3.99 |
| **Mortality** | 0.00436 – 0.0131 | 3.82 – 3.87 |

(B)

|  | **Range Swept** | **Resulting Average Duration (months)** |
| --- | --- | --- |
| **Treatment Admission** | Time-varying | 3.83 – 5.89 |
| **Moderate Wasting Recovery** | 0.0391 - 0.117 | 3.86 - 5.59 |
| **Severe Wasting Incidence** | 0.0263 - 0.0939 | 4.36 – 5.77 |
| **Severe Wasting Recovery** | 0.0457 – 0.137 | 4.55 – 4.69 |
| **Defaulting** | 0.00221 – 0.00663 | 4.61 - 4.68 |
| **Mortality** | 0.00130 – 0.00390 | 4.62 – 4.67 |

**Table S1.** Range of values swept and resulting average model-derived duration of episode found from sensitivity analysis. **(A)** Sensitivity analysis results for severe wasting incidence. **(B)** Sensitivity analysis results for moderate wasting incidence.
